# Supplementary material for: Enhanced Thermal Conductivity of Silicone Composites Filled with Few-Layered Hexagonal Boron Nitride
Source: Polymers (Basel). 2020 Sep 12;12(9):2072. doi: 10.3390/polym12092072 (PMC7570332; doi:10.3390/polym12092072)
Supplement: Supplementary file 1 [file polymers-12-02072-s001.pdf]

Supporting Information

Enhanced thermal conductivity of silione composites filled with few-layered hexagonal boron nitride

Wei-Cheng Cheng, Yi-Ting Hsieh and Wei-Ren Liu*

Department of Chemical Engineering, R&D Center for Membrane Technology, Research Center for Circular Economy, Chung Yuan Christian University, 200 Chung Pei Road, Chungli District, Taoyuan City, 32023, Taiwan, R.O.C.

**E-mail address: WRLiu1203@gmail.com*

Tel:+886 3-265-4140; fax: +886 3-265-4199

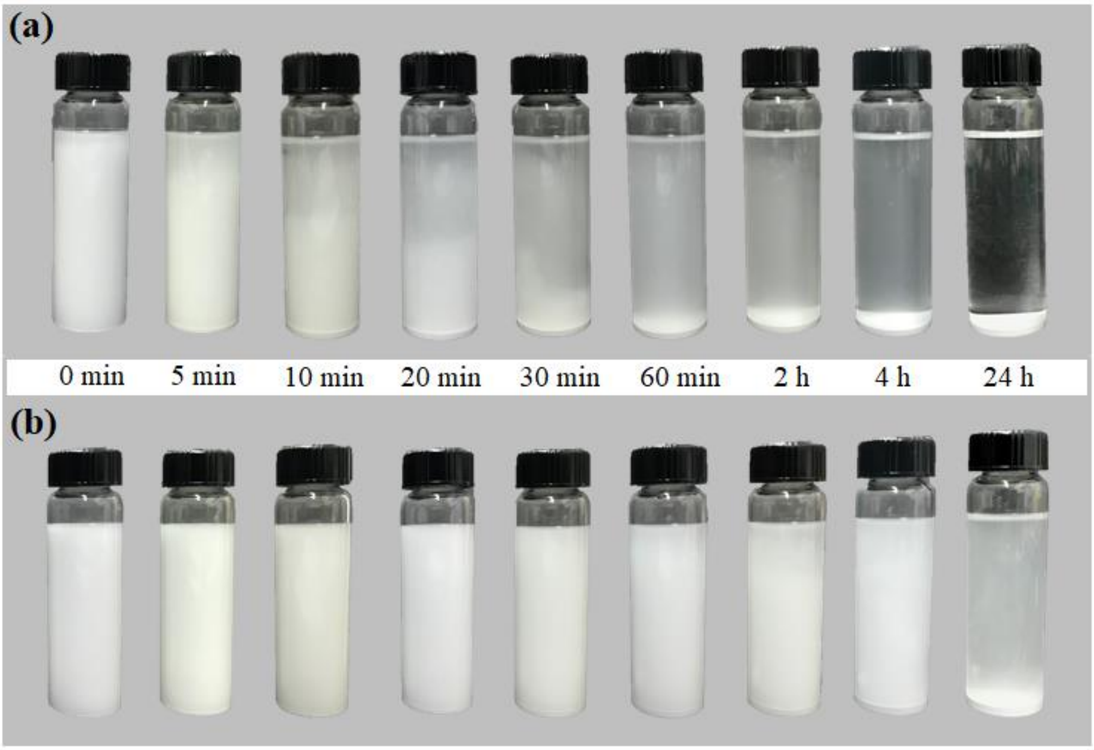

Figure S1. Photo images of (a) pristine h-BN and (b) FL-hBN solution after different resting time.

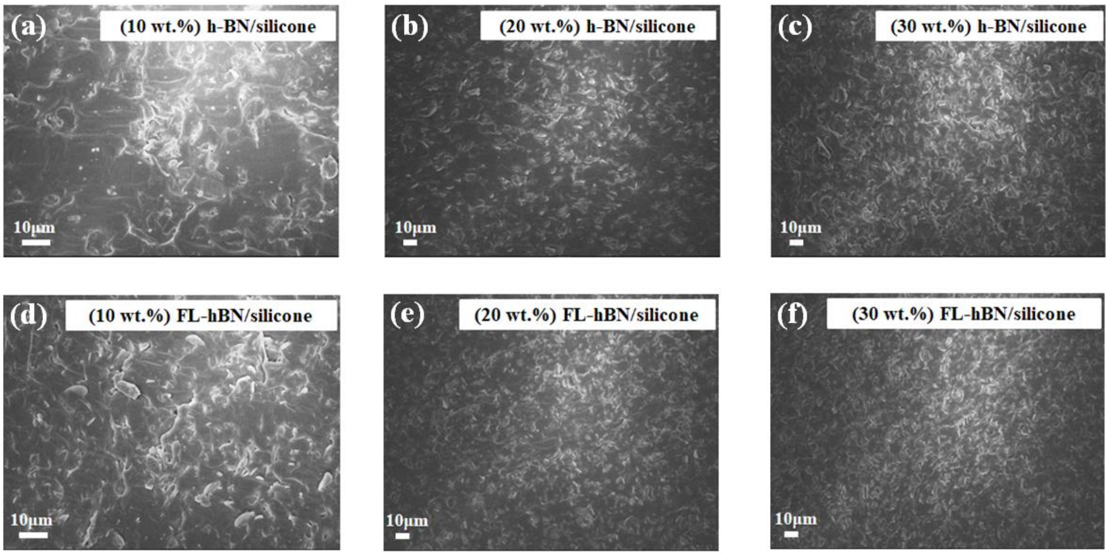

Figure S2. (a)-(c) SEM images of h-BN/silicone composites with powder loading of 10 wt%, 20 wt%, 30 wt% ; (d)-(f) SEM images of FL-hBN/silicone composites with powder loading of 10 wt%, 20 wt%, 30 wt%.

Table S1. FWHM, Lc and La of h-BN and FL-hBN.

	**FWHM(002)(°)**	**Lc (002)(Å)**	**La (100)(Å)**
**h-BN**	0.3191	256.2	228.8
**FL-hBN**	0.4130	197.9	213.5
